# Mothers’ psychological distress during COVID-19 pandemics: three-waves longitudinal study

**DOI:** 10.1186/s40359-025-02587-2

**Published:** 2025-03-24

**Authors:** Fitri Ariyanti Abidin, Laila Qodariah, Vidya Anindhita, Fitriani Yustikasari Lubis, Zahrah Aulianissa Manindjo, Fredrick Dermawan Purba

**Affiliations:** 1 Department of Psychology, Faculty of Psychology, Universitas Padjadjaran, Sumedang, Indonesia; 2 Center for Relationship, Family Life and Parenting Studies, Faculty of Psychology, Universitas Padjadjaran, Sumedang, Indonesia; 3 Center for Psychological Intervention and Research, Faculty of Psychology, Universitas Padjadjaran, Sumedang, Indonesia

**Keywords:** COVID-19, Mothers, Depression, Anxiety, Stress, Longitudinal study

## Abstract

**Background:**

The COVID-19 pandemic has profoundly impacted the psychological well-being of populations worldwide. Despite this, there is a paucity of research on the specific psychological distress experienced by mothers during this crisis. This study aims to address this gap by examining the trajectories of psychological distress experienced by Indonesian mothers during the COVID-19 pandemic.

**Methods:**

A sample of 108 mothers aged 25 to 65 (mean = 38.9, SD = 7.3) participated in three waves of data collection during the lockdown phase, adaptation phase, and new normal phases of the pandemic. Participants completed the Indonesian version of the Depression, Anxiety, and Stress Scale-18 (DASS-18) questionnaire to assess their levels of depression, anxiety, and stress.

**Results:**

Depression remained constant while anxiety and stress levels decreased over time. Notably, older participants reported lower levels of stress than their younger counterparts, and those who had been married for a longer time reported lower levels of stress.

**Conclusion:**

This study provides critical insights into the mental health status of Indonesian mothers during the COVID-19 pandemic, highlighting the importance of considering contextual factors such as age and length of marriage in interventions and support programs.

## Background

The outbreak of COVID-19 has significantly impacted multiple facets of human life, including mental health [1, 2]. Along with its physical consequences, the pandemic has caused devastating effects on individuals’ mental well-being. To mitigate the spread of the virus and allow time for public health officials and scientists to gain a better understanding of the virus, develop treatments and vaccines, and enhance healthcare systems’ responsiveness, nearly all nations worldwide have implemented lockdown policies. This has resulted in the closure of schools, higher education institutions, workplaces, and the shutting down of borders, creating a situation that necessitates quick adaptation. Consequently, remote work and distance learning have become the new societal norms [3].

The unprecedented situation has significantly affected families, who reside, work, and study in the same physical space. Education has shifted to the home environment, and parents and caregivers have taken up the responsibility of both educating children and adjusting to the extensive changes necessitated by lockdown measures. For many families, these changes have resulted in financial stressors, limited space for work and study, heightened childcare demands, and work-family conflicts. Women have been particularly impacted by these circumstances [4–8].

Previous research suggests that mothers have been taking on an increasingly larger share of the added childcare and home-based schooling responsibilities [9–11]. In Indonesia, the influence of patriarchal norms within society [12] tends to delineate distinct roles in childcare and domestic management between genders [13]. Most Indonesian mothers bear a disproportionate childcare burden than fathers in daily life [14]. They are predominantly tasked with the nurturing, guidance, and provision of attentive care infused with affection for their children [15]. The holistic fulfillment of children’s needs—physical, psychological, social, and spiritual—is seen as a fundamental aspect of maternal duty [16]. This dynamic exacerbates the challenges faced by Indonesian mothers amid the COVID-19 pandemic. They have encountered difficulties in juggling work, household chores, caregiving, and facilitating home-based schooling [17].

Multiple lines of evidence suggest that the increased stress and responsibilities experienced by mothers have had a significant impact on their psychological well-being. For instance, Goyal et al. [18] discovered that mothers of young children reported elevated levels of stress and anxiety during the early stages of the pandemic. Furthermore, mothers were found to be at a greater risk of depression during the pandemic, particularly evident among women who experienced economic struggle, encountered challenges in reconciling homeschooling demands with work responsibilities, and had less access to childcare services [19]. Disruption of maternal-infant bonding has been reported in many mothers due to heightened levels of anxiety and stress during the caregiving process within an inadequate environmental setting [20]. In addition, the study by Collins et al. [21] revealed that along with the closure of school and daycare, working mothers with young children at home reported a significant reduction in their work hours compared to fathers. This finding highlights the likelihood of mothers encountering greater challenges in balancing their work and caregiving responsibilities during the pandemic.

Despite several studies on mothers’ psychological distress, the majority of these results were obtained from cross-sectional designs. Therefore, a longitudinal approach to comprehensively describe the patterns of change and development of mothers’ psychological distress remains limited. The available longitudinal literature concerning psychological distress primarily focuses on the general adult population [22–24], university students [25], employees [26], teachers [27], or a specific vulnerable population, such as primary school children and patients with eating disorders [28, 29]. Those studies reveal varying results. Some studies highlight an improvement in mental health over time [22, 30]. Some longitudinal studies suggest stability in mental health status [24]. However, more studies indicate a worsening of mental health [23, 25–27, 31–33].

Although the COVID-19 pandemic may be over or controlled to some extent, its psychological effects can persist long after the immediate threat has subsided. This is particularly evident in the concept of COVID-19 Anxiety Syndrome, which is characterized by maladaptive behaviors such as avoidance, excessive threat monitoring, and pervasive worry. Research indicates that individuals exhibiting high levels of this syndrome are more likely to experience prolonged psychological distress, including heightened anxiety and depression, as these coping mechanisms interfere with the natural extinction of fear responses [34]. Given these long-term mental health implications, it is crucial to identify and address ongoing psychological challenges. Continued research into the mental health impacts of the pandemic will provide a deeper understanding of these issues and inform the development of effective interventions and policies to support affected individuals.

Building upon this premise, the present study aims to investigate the trajectories of psychological distress experienced by Indonesian mothers during the different phases of the COVID-19 pandemic, including the lockdown phase, adaptation phase, and new normal phase.

## Methods

### Participants

A sample of 108 mothers, aged between 25 and 65 years old, participated in the present study with a mean age of 38.9 (SD = 7.3). These participants had been married for a period ranging from 3 to 36 years, with a mean duration of 12.9 years. The mothers had an average of two children, with the number of children ranging from 1 to 7. The majority of the participants were married (92.6%), while the remaining were single mothers. With respect to employment status, the majority of participants were housewives (49.1%), followed by working mothers who worked from home (40.7%), with the remainder of the participants being working mothers who worked outside of the home. Furthermore, most of the mothers had a university degree (95.4%). Detailed information is provided in Table 1.

**Table 1:** Demographics characteristics of participants

Variable	*n* (%)
Age	
25–35	35 (32.41%)
36–45	57 (52.78%)
46–65	16 (14.81%)
Spouse’s Age	
27–35	28 (26.92%)
36–45	51 (49.04%)
46–66	25 (29.04%)
Marriage Duration	3–36
3–10	38 (35.19%)
11–20	56 (51.85%)
21–36	13 (12.04%)
Number of Children	
1–3	98 (90.74%)
4–7	10 (9.26%)
Marriage Status	
Married	100 (92.6%)
Single Mother	4 (3.7%)
Long Distance Marriage	4 (3.7%)
Working Arrangement	
Housewife	53 (49.1%)
Work-from-home	44 (40.7%)
Shift	9 (8.3%)
Work-from-outside	2 (1.9%)
Educational Background	
University	103 (95.4%)
Diploma	4 (3.7%)
High School	1 (0.9%)

The participants for this study were recruited through social media platforms such as Facebook, Instagram, and Twitter. We disseminated an invitation to participate in the study, along with the informed consent and survey link. Interested and eligible participants were able to access the survey directly through the provided link. Prior to the commencement of the online survey, informed consent was obtained from all participants.

### Procedures

Psychological distress was measured in the same group of participants at three time points: Wave 1 (April 20—25, 2020), Wave 2 (May 19—August 31, 2020), and Wave 3 (October 29—November 15, 2021). These three waves represented different phases in the Indonesian government and people during the COVID-19 pandemic. We followed the stringency index, which is a composite measure based on nine response indicators including school closures, workplace closures, and travel bans, rescaled to a value from 0 to 100 (100 = strictest) (see https://ourworldindata.org/explorers/covid?uniformYAxis=0%26country=~IDN%26hideControls=true%26Metric=Stringency+index%26Interval=7-day+rolling+average%26Relative+to+population=true). The first wave was measured during the peak of the stringency index (lockdown) of Indonesia. The second wave was the time that Indonesian people adapted to COVID-19-related regulations (stringency index was decreasing). The third wave was the phase when Indonesians were living a ‘new normal’ life with COVID-19 vaccination already available (low stringency index). In each wave, the participants completed the e-survey in 10–20 min.

### Instruments

Participants were asked questions regarding their demographic background (age, educational level, working status) and their family (age of marriage, age of their spouse, number of children).

### Depression anxiety stress scale – 18 (DASS-18)

Psychological distress was measured using the Indonesian version of the Depression Anxiety Stress Scale-18 (DASS-18), which is recommended for use in Asian samples [35]. This version is adapted from the original DASS-21 [36]. DASS-18 is a self-report questionnaire that consists of 18 items: depression (7 items), anxiety (7 items), and stress (4 items). Participants were asked to rate their condition by using 4 Likert scales: 0 = do not suit me at all or never; 1 = agree with me to some degree or sometimes; 2 = agree with me to a considerable extent, or often; 3 = it suits me very well or almost always. The internal consistency of the DASS-18 was excellent across the waves: Wave 1 (α = 0.927), Wave 2 (α = 0.923), and Wave 3 (α = 0.940). For the subscales, Depression had α = 0.846 (Wave 1), 0.866 (Wave 2), and 0.902 (Wave 3); Anxiety had α = 0.849 (Wave 1), 0.848 (Wave 2), and 0.845 (Wave 3); and Stress had α = 0.805 (Wave 1), 0.727 (Wave 2), and 0.827 (Wave 3).

### Statistical analysis

Statistical analysis was conducted using SPSS Statistics software (version 24). Demographic data were analyzed by calculating the range, central tendency, and percentage. The normality of continuous variables was assessed using the Kolmogorov-Smirnov test, which indicated that all data were not normally distributed. Therefore, Spearman’s correlation was employed to examine the relationship between demographic characteristics (length of marriage, number of children, and age) and psychological distress in each wave. A correlation coefficient of 0.70–0.90 was considered strong, 0.40–0.60 moderate and 0.10–0.30 was considered weak [37]. The Friedman test was used to determine differences in depression, anxiety, and stress scores across waves. When a significant difference was found, post hoc analysis was conducted using the Bonferroni technique. Differences between working and non-working mothers in terms of depression, anxiety, and stress in each wave were assessed using the nonparametric Mann–Whitney U test.

## Results

**Fig. 1:**
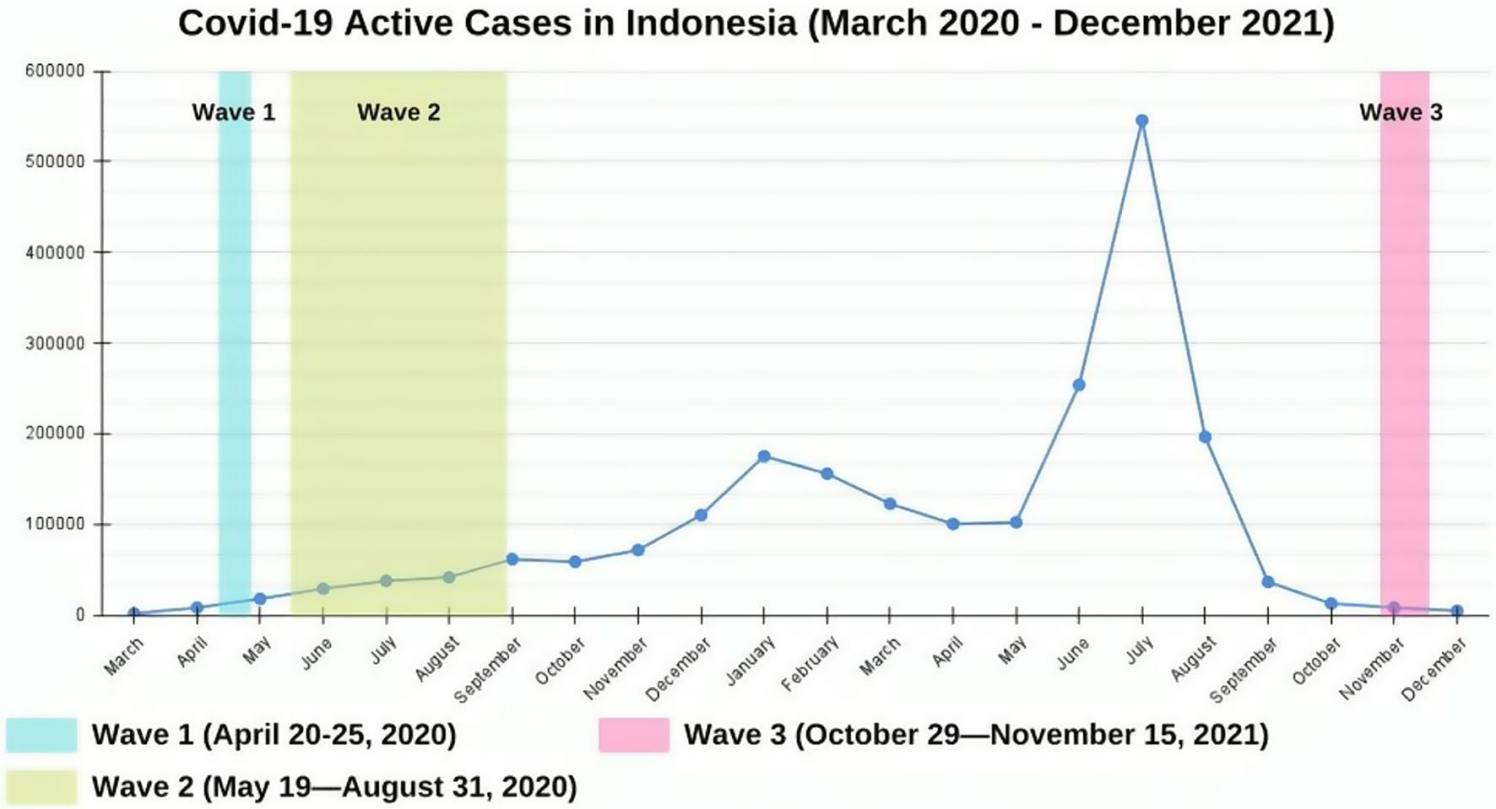
The graph of COVID-19 active cases in Indonesia during data collection

The first and second data collection were conducted in the early phase of the outbreak (April 20–August 31, 2020), while the third data collection was conducted after the huge strike of new cases (October 29—November 15, 2021). The COVID-19 active cases in Indonesia during the data collection are presented in Fig. 1.

The statistical analysis in Table 2 showed a significant difference in the psychological distress score and anxiety score between Wave 2 and Wave 3; participants’ psychological distress and anxiety in Wave 2 were significantly higher than in Wave 3. As for the stress score, significant decreases were found between Wave 1 and Wave 3. Thus, we can conclude that there was a significant decreasing trend in the level of one’s psychological distress, anxiety, and stress over time.

**Table 2:** Descriptive statistics and difference test of DASS, depression, anxiety, and stress in three waves

	Wave 1 April 2020	Wave 2 May-Aug 2020	Wave 3 Nov 2021	χ^2^	*p*	Post hoc
Depression	1.41 (0.47)	1.42 (0.49)	1.36 (0.51)	5.90	0.052	
Anxiety	1.55 (0.53)	1.57 (0.54)	1.47 (0.49)	9.39	0.01	Wave 2- Wave 3
Stress	1.84 (0.59)	1.79 (0.53)	1.72 (0.60)	8.18	0.017	Wave1- Wave 3

Table 3 presents the correlations between demographic characteristics and depression, stress, and anxiety during the three waves of data collection. Both age and length of marriage are negatively associated with depression, anxiety, and stress, meaning that older individuals and those in longer marriages tend to experience lower levels of psychological distress. However, length of marriage generally shows stronger and more consistent correlations, particularly in Wave 1. Length of marriage also maintains a sustained negative relationship with stress across all waves. The number of children, however, shows a less consistent relationship with psychological distress.

**Table 3:** Spearman correlation analysis between demographic characteristics and the main variables (depression, anxiety, stress) in three waves

Variables	Skewness	Kurtosis	1	2	3	4	5	6	7	8	9	10	11	12
1. Length of Marriage			-											
2. Number of children			0.428**	-										
3. Age			0.829**	0.390**	-									
4. Depression Wave 1	1.87	4.36	− 0.310**	− 0.125	− 0.291**	-								
5. Anxiety Wave 1	2.18	5.97	− 0.205*	− 0.102	− 0.183	0.619**	-							
6. Stress Wave 1	2.44	7.51	− 0.337**	− 0.127	− 0.298**	0.634**	0.693**	-						
7. Depression Wave 2	1.93	4.26	− 0.153	− 0.196*	− 0.189*	0.639**	0.579**	0.597**	-					
8. Anxiety Wave 2	1.95	4.70	− 0.184	− 0.104	− 0.248**	0.538**	0.705**	0.558**	0.607**	-				
9. Stress Wave 2	1.58	2.77	− 0.260**	− 0.066	− 0.285**	0.652**	0.616**	0.707**	0.720**	0.687**	-			
10. Depression Wave 3	0.46	-0.09	− 0.159	− 0.262**	− 0.108	0.495**	0.403**	0.438**	0.648**	0.451**	0.461**	-		
11. Anxiety Wave 3	0.46	-0.09	− 0.314**	− 0.134	− 0.248**	0.444**	0.527**	0.508**	0.475**	0.631**	0.462**	0.609**	-	
12. Stress Wave 3	0.91	0.78	− 0.269**	− 0.133	− 0.213*	0.473**	0.452**	0.624**	0.488**	0.543**	0.515**	0.695**	0.751**	-

Note. ** *p* <.05, * *p* <.01

Table 4 shows a comparative analysis of depression, anxiety, and stress levels among non-working and working mothers. The results indicated that in Wave 1, non-working mothers had significantly greater levels of depression than working mothers, z = [-2.00], p = [0.05]. In wave 3, non-working mothers also had significantly greater levels of stress than their counterparts, z = [-2.29], p = [0.02].

**Table 4:** Comparative analysis of depression, anxiety, and stress levels among non-working and working mothers

	Non-working mothers M (SD)	Working Mothers M (SD)	Mann-Whitney	
			z	p
Depression Wave 1	1.51 (0.54)	1.32 (0.36)	**-2.00**	**0.05**
Anxiety Wave 1	1.63 (0.59)	1.46 (0.45)	-1.56	0.12
Stress Wave 1	1.94 (0.70)	1.75 (0.46)	-0.76	0.45
Depression Wave 2	1.47 (0.59)	1.36 (0.37)	-0.66	0.51
Anxiety Wave 2	1.64 (0.61)	1.51 (0.45)	-0.87	0.38
Stress Wave 2	1.84 (0.57)	1.75 (0.49)	-0.86	0.39
Depression Wave 3	1.42 (0.60)	1.30 (0.39)	-1.27	0.21
Anxiety Wave 3	1.54 (0.56)	1.41 (0.40)	-1.03	0.30
Stress Wave 3	1.84 (0.63)	1.60 (0.54)	**-2.29**	**0.02**

## Discussion

The aim of this study was to investigate the trajectories of psychological distress experienced by Indonesian mothers during different phases of the COVID-19 pandemic, namely, the lockdown, adaptation, and new normal phases. The results of our study demonstrated that depression remained stable while anxiety and stress decreased over time. These findings are partially consistent with previous research reporting stability or reduction in the levels of depression, anxiety, and stress. Specifically, our findings regarding the sustained state of depression align with Wang’s [24] investigation of mental health outcomes in the general Chinese population, which reported no significant longitudinal changes in these psychological constructs. Moreover, the decline in anxiety and stress observed in our study is similar to Zhou et al.‘s [30] investigation of anxiety and stress in the adult population of the United States.

Our findings differ from Planchuelo-Gómez’s [23] survey of the adult population, which reported significant increases in symptomatic scores of depression, anxiety, and particularly stress. Similarly, Th’ng et al. [38] also found a significant increase in depression among healthcare workers in Singapore, while Zhang et al. [25] found an increased trend in anxiety among college students. The differences in epidemiological situations between these studies may contribute to the disparate findings. Notably, while the peak cases were experienced between the second and third waves of data gathering in our study, the third wave was collected a year after the second wave. It is plausible that the mothers in our sample had developed effective coping strategies to overcome their stress and anxiety over time. Research has shown that coping strategies can be effective in reducing the negative impact of the COVID-19 pandemic on mental health [39–41].

The Resiliency Model of Family Stress, Adjustment, and Adaptation offers insight into the decrease in maternal stress and anxiety during the second wave of the COVID-19 pandemic [42]. According to this model, when a family encounters a crisis marked by “imbalance, disharmony, and disorganization in the family system,” as observed in this study regarding the stressor linked to the pandemic, mothers undergo two clear stages: adjustment and adaptation. To tackle this crisis, mothers play an active role in the adjustment phase by engaging in situational appraisal, fostering positive mindsets, and seeking out social support.

Social support from spouses and other family members significantly enhances Indonesian mothers’ ability to cope with stressful situations, as evidenced by various studies [43–46]. This support may involve sharing responsibilities or providing financial assistance to meet their needs. Acting as a buffer, social support helps alleviate stress and influences parents’ coping strategies by offering both practical information and emotional reinforcement [47]. These coping mechanisms reflect Indonesia’s collectivist culture. Another distinctive coping mechanism in Indonesia, compared to Western countries, is religious coping [43, 48, 49], which fosters gratitude and positive interpretations of stressful situations [50]. This mindset may facilitate better management of challenging circumstances, a key aspect of parental resilience [51]. Additionally, patience and sincerity in facing challenges have emerged as significant factors in adaptive evaluation in previous Indonesian studies. A positive outlook enables parents to cultivate compassion, reduce selfishness, and enhance caregiving and mindfulness, all of which contribute to positive parenting [45, 52]. Consequently, parents transition to the adaptation stage, where they analyze the situation, adjust to patterns of change, employ problem-solving and coping strategies, and demonstrate resilience by bouncing back and adapting in crisis situations. Our study identified age and length of marriage are associated with stress levels. Specifically, we found that older participants had lower stress levels, while younger participants reported higher stress levels. These findings are consistent with previous research demonstrating a negative relationship between age and psychological distress, as reported by Gutiérrez-Hernández et al. [53], Justo-Alonso et al. [54], and Huang & Zhao [55]. One possible explanation for our results could be the lack of adequate social contact during the pandemic, which younger people may be more dependent on as a source of support and information compared to older people, as highlighted by Gutiérrez-Hernández et al. [53]. Moreover, the uncertainties caused by the pandemic may have a greater impact on the academic, career, social, and economic prospects of younger people who are still in the early stages of pursuing these goals [53]. Additionally, younger people may experience a lack of daily routine during the pandemic, since their regular activities outside of the home are often interrupted [54].

The study also indicated that individuals who had a longer duration of marriage reported lower levels of stress. The correlation between the length of the marriage and lower levels of stress found in the study may suggest that individuals who have been in long-term marriages may have developed stronger coping mechanisms, social support networks, and a greater sense of stability and security in their relationships. These factors may help to mitigate the stress and anxiety associated with the COVID-19 pandemic, as individuals with stronger social support networks and coping mechanisms may be better equipped to deal with the challenges posed by the pandemic [56]. Our findings suggest another potential explanation: Younger mothers with shorter marriage durations may have young children who require greater attention and are more dependent, whereas older mothers with longer marriages may have more independent children. Parenthood typically brings about time constraints, leading to increased stress and decreased well-being, particularly when children are younger [57].

In the lockdown phase, our study revealed a notable disparity in stress and depression levels between non-working and working mothers. Specifically, non-working mothers exhibited significantly higher levels of stress and depression compared to their working counterparts. This discrepancy highlights the potential psychological benefits of involvement in work-related tasks, providing a source of personal satisfaction for working mothers despite the inherent challenges and pressures in the workplace [58]. The lockdown, characterized by reduced work hours and flexible work arrangement [21], allows working mothers to allocate more time to addressing family responsibilities and personal well-being [59]. The decrease in employment demands, possibly to accommodate rising domestic responsibilities and childcare duties during the pandemic, may have been more acceptable in the workplace compared to pre-pandemic times. Consequently, this flexibility enables them to maintain a balance between work and family duties, contributing to an increase in personal satisfaction and happiness [60]. Furthermore, full-time employment provides financial stability and increase economic resilience [61], serving as a pivotal protective factor for the well-being of mother. Additionally, employment provides opportunities for meaningful engagement, both through paid work and spending quality time with children, diverting attention away from the pandemic and reduce work-family conflict [62]. This further adds to the multifaceted benefits posed by employment status for mothers, underscoring possible coping mechanisms during unprecedented times such as the pandemic, as identified in our study.

One major strength of this study is that it is the first longitudinal study to investigate the trajectories of psychological distress among Indonesian mothers during the COVID-19 pandemic. This approach allows for the examination of changes in psychological distress over time and provides a more nuanced understanding of the mental health impacts of the pandemic. One limitation of the present study is that the assessment of depression, anxiety, and stress levels relied solely on self-report measures, which are subject to response biases and may not accurately reflect the participants’ true psychological state. Additionally, the study collected data using an online platform, which may have introduced sampling biases and limited the generalizability of the results to individuals who have access to the internet and are comfortable using digital devices. Furthermore, the study’s sample size was relatively small, and participants were recruited from a single province in Indonesia, which may limit the generalizability of the findings to other regions or countries. Future studies should consider using multiple assessment methods and recruiting larger and more diverse samples to address these limitations. Additionally, no formal power analysis was conducted to determine the optimal sample size, increasing the risk of Type II errors and limiting the reliability of the results. Future studies should conduct power analyses, use multiple assessment methods, and recruit larger, more diverse samples to enhance the validity and generalizability of the findings. Another limitation is the inability to control for covariates when examining changes in psychological distress across waves. While ANCOVA/MANCOVA could provide a more precise analysis by adjusting for prior wave scores, these methods require normality and homogeneity of variances, which were not met in our dataset. Instead, we employed the Friedman test, a nonparametric alternative to repeated-measures ANOVA, which does not allow for the inclusion of covariates. As a result, potential confounding effects could not be statistically controlled. Future research with larger and more normally distributed samples may consider ANCOVA/MANCOVA to account for prior wave scores and enhance precision. Lastly, the current study did not gather data on pre-existing health conditions, health status across the three measurement points, illness history, or mental health history. Future research should prioritize the collection of such data to control for pre-existing mental health issues.

## Conclusions

The results of this study have important implications for healthcare practitioners and policymakers. First, the finding that depression remained steady while anxiety and stress decreased over time may reflect the natural adjustment or resilience that developed among mothers during the pandemic, rather than the result of specific psychological interventions or formal support. This highlights the complex nature of maternal mental health, where certain dimensions of distress (like anxiety and stress) may be more responsive to changing circumstances, while others (like depression) may be more resistant and demand sustained attention.

Second, the negative correlation between age and stress levels, as well as the negative correlation between length of marriage and stress levels, suggests that older and more experienced mothers may be better equipped to cope with stressors compared to younger or less experienced mothers. This information could inform the development of targeted interventions for mothers who may be more vulnerable to stressors, such as younger or less experienced mothers. Overall, the findings of this study provide important insights into the mental health conditions of Indonesian mothers during an unprecedented stressor and highlight the importance of providing timely and appropriate psychological support to mothers during crises. The study’s results could inform the development of targeted interventions for mothers who may be more vulnerable to stressors and ultimately help improve the mental health and well-being of mothers during future crises.

## Data Availability

The datasets used and analyzed during the current study are available from the corresponding author upon reasonable request.

## Abbreviations

ANCOVA: Analysis of Covariance
COVID-19: Coronavirus Disease 2019
DASS: Depression, Anxiety, and Stress Scale
MANCOVA: Multivariate Analysis of Covariance
SD: Standard Deviation
SPSS: Statistical Package for the Social Sciences
